# Preparation of biosorbent from avocado seeds for preconcentration and simultaneous extraction of trace parabens in environmental wastewater samples

**DOI:** 10.3389/fchem.2025.1688983

**Published:** 2025-11-27

**Authors:** Aluwani Sharon Nephiphidi, Rabelani Mudzielwana, Shirley Kholofelo Selahle

**Affiliations:** 1 Department of Chemistry, Faculty of Science, Engineering and Agriculture, University of Venda, Thohoyandou, South Africa; 2 Department of Geography and Environmental Science, Faculty of Science, Engineering, and Agriculture, University of Venda, Thohoyandou, South Africa

**Keywords:** avocado seeds, biosorbent, dispersive magnetic solid-phase microextraction, magnetic carbon activated, parabens

## Abstract

The identification of emerging contaminants, including parabens, in water resources and aquatic ecosystems has become an issue of environmental concern due to the associated human and ecological risks, emphasizing the urgent need for effective monitoring and remediation strategies. This study aimed to synthesize a low-cost magnetic activated carbon biosorbent from avocado seeds, achieving higher extraction efficiencies and paving the way for more sustainable water treatment methods. The Dispersive Magnetic Solid-Phase Microextraction (DMSPME) technique was used for the preconcentration and simultaneous extraction of parabens (methylParaben, ethylParaben, and butylParaben). Influential DMSPME parameters were optimized using a Fractional factorial design for screening and a central composite design for further analysis. Characterization results have proved that the desired biosorbent was successfully synthesized. The magnetic activated carbon showed a well-defined surface with exhaustive characterization of Brunauer-Emmett-Teller (BET) revealed surface areas of 30 m^2^/g and a pore volume of 0.1 cm^3^/g. The exhaustive characterization of the Scanning Electron Microscope (SEM) image revealed that the incorporation of iron oxide into the activated carbon resulted in the formation of small, well-defined pores. The Energy Dispersive X-Ray (EDS) indicated the existence of carbon, oxygen, iron, sodium, and potassium. The Fourier-Transform Infrared Spectroscopy (FTIR) analysis revealed the presence of several functional groups, including carboxylic acid, iron oxide, and aromatic compounds. This characterization confirmed that Magnetic activated carbon prepared with 30% KOH was the best biosorbent in extracting the targeted parabens. The parameters of interest were biosorbent amount, sample volume, type of elution solvent, pH, and volume of extraction solvent. The optimum parameters obtained were 22.5 mg of 30% KOH-magnetic carbon activated, 6.5 pH, and 875 µL methanol as elution solvent. These conditions lead to obtaining good relative recoveries of 80.0%–101%. Limit of detection (LOD) and Limit of quantification (LOQ) of the method were 0.12–0.19 μg/L and 0.25–0.49 μg/L, respectively. The reusability and regeneration adsorption percentage recoveries significantly decreased after the fifth cycle. Parabens extraction using the 30% KOH- magnetic activated carbon was considered a capable biosorbent for the extraction of parabens because it demonstrated a strong potential for extracting parabens pollutants simultaneously in wastewater samples.

## 1 Introduction

Water is an essential resource for economic activity, environmental balance, and human survival. Water is required in various industries, the agricultural sector, for drinking, and to support ecosystems. However, public health and water security are at risk due to the growing number of pollutants contaminating water bodies. Parabens, or para-hydroxybenzoates, are one of these contaminants that have drawn the most attention due to their frequent detection in aquatic environments (Derisso et al., 2020). They find their way into water bodies via household wastewater, including improper disposal of goods and industrial discharge from manufacturing operations (Vale et al., 2022). Parabens are created synthetically by esterifying p-hydroxybenzoic acid (pHBA) with an alcohol in the presence of a catalyst (Morelli et al., 2020). These compounds share a similar chemical structure, featuring a benzene ring, a hydroxyl group, and an ester group at the para position on the ring. However, they vary in their substituents, which can be either an aromatic ring or an alkyl chain. The most utilized parabens are: methylparaben (MethylP), ethylparaben (EthylP), propylparaben (PropylP), and butylparaben (ButylP) (Sousa et al., 2020).

Parabens are commonly used as preservatives in food products, pharmaceuticals, and cosmetics. Due to the widespread use of parabens as the most primarily used chemical, they have been found in water bodies in concentrations varying from ng/L to µg/L (Wei et al., 2021). Parabens concentrations vary depending on location, water type, and wastewater treatment efficiency. Supplementary Table S1 lists the various concentrations obtained from several countries. In Africa, parabens were detected in South Africa and Nigeria (Leung et al., 2024; Mpayipheli et al., 2024). In both countries, they were detected in wastewater and surface water, with concentrations ranging from non-detectable to 84.7 μg/L and non-detectable to 0.52 mg/L, respectively. In Asia, the concentrations of China’s surface water and India Lake water were reported to be 8.38 ng/L and 0.0182 mg/L (Kachhawaha et al., 2021; Zhao et al., 2024). In Europe, parabens have been detected in Switzerland’s Glatt River at higher concentrations (Lincho et al., 2021). The global concentration of parabens suggests that traces of these compounds are found worldwide, making the contamination of water bodies by parabens a global concern.

The most stressful problem is that parabens can be active in very low concentrations and can interact with any other organic compounds present in the water bodies (Derisso et al., 2020), and there is no prediction of what might happen after the interaction. Al-Qudaihi et al. (2025) demonstrated that the Acceptable Daily Intake (ADI) for combined intake of MethylP and EthylP, as assisted by the European Food Safety Authority (EFSA) and JECFA, is 0–10 mg/kg. As for Butyl, it was not accepted as a food additive or any other consumable additive according to the Scientific Committee on Consumer Safety (SCCS) (Bernauer et al., 2023). From the European Union, it could only be used in cosmetic products with a concentration not exceeding 0.028% in the final product because it was found not to be safe for children (0–10 years) when combined with other chemicals (Gaffet et al., 2025). Due to their endocrine-disrupting properties, parabens are associated with several adverse health effects. They can mimic estrogen and disrupt hormonal balance, which may have an impact on puberty, fertility, and reproductive development. Reduced sperm quality in men and early puberty or reproductive problems in women and children have been related to elevated levels of chemicals such as butylparaben and methylparaben. Concerns regarding parabens’ possible involvement in hormone-related cancers have been raised by their detection in human tissues, including tumors (Liang et al., 2023). They can also result in allergic reactions and skin irritation, especially in people with sensitive or damaged skin (Le et al., 2022).

To mitigate these risks, adsorption-based techniques have proven to be a promising approach for extracting parabens, thereby enhancing their detection and facilitating water treatment efforts. Agricultural waste, such as nutshells (Narloch et al., 2024), plum shells (Moreno-Marenco et al., 2022), grapefruit peels (Molina-Balmaceda et al., 2025), and cork pellets (Morelli et al., 2020), has proven to be a promising source for producing carbon absorbents for extracting parabens in aqueous solutions. Avocado seeds have also been tested as an adsorbent for organic contaminants from water. Its effectiveness in adsorbing various pollutants is attributed to the bioactive compounds it contains. These bioactive substances include procyanidins, phenolic compounds, triterpenoids, acetogenins, fatty acids, amines, and weak acids, among others (García-Vargas et al., 2020). These Avocado seeds have also proven to be a great source of activated carbon for various applications, such as the adsorption of gases and the removal of contaminants in the environment (Ahmad and Danish, 2022). Siemak and coworkers (Siemak and Michalkiewicz, 2024) demonstrated that avocado seed activated carbon (ASAC) has a high specific surface area and is efficient in extracting organic compounds such as ibuprofen (Molina-Balmaceda et al., 2024), dyes (Munyegaju, 2022), and Phenols (Kaga et al., 2024). In addition, Riadi et al. further supported this, showing ASAC is effective for extracting dyes from water (Riadi et al., 2023). These studies collectively demonstrate ASAC’s efficacy as an adsorbent for various emerging pollutants in water.

The research focuses on the development of magnetic activated carbon (MAC) as an improved version of traditional activated carbon, which often faces challenges with complex separation techniques (Zakaria et al., 2023). By incorporating magnetic properties, MAC enhances efficiency and simplifies the recovery process from treated water, thereby improving the material’s reusability. MAC is created by coating activated carbon with iron salts, such as FeCl_3_ and FeSO_4_, followed by the addition of a base to precipitate magnetic iron oxide. This study also examines the innovative use of MAC derived from avocado seeds to extract and concentrate parabens (methylP, ethylP, and butylP) from wastewater. The valorization of avocado seed waste not only transforms it into a valuable resource, promoting recycling and reusing, but also contributes to the circular economy by optimizing the use of natural resources for water treatment (Okuthe, 2024). The low-cost, eco-friendly material offers higher extraction efficiencies of the targeted analytes, earning it the designation of biosorbent and highlighting the uniqueness of the research. While previous studies have utilized avocado seed waste for carbon-based absorbents, few have focused on modified carbon for extracting parabens from wastewater. This study employs Dispersive magnetic solid-phase micro extraction (DMSPME) as an advanced method for extracting contaminants. Using a magnetic bio sorbent for the simultaneous extraction of parabens builds upon previous research. The effectiveness of this technique is underscored by high-performance liquid chromatography with a photodiode array detector (HPLC-PDA), which confirms high recovery rates and demonstrates the practical applicability of the developed DMSPME method for environmental monitoring.

## 2 Experimental

### 2.1 Chemicals and reagents

Potassium hydroxide (KOH 98%), ferrous sulphate (FeSO_4_·7H_2_O 98%), sodium hydroxide (NaOH>98%), Ferric chloride (FeCl_3_.6H_2_O 28%), and hydrochloric acid (HCl >95%) were all purchased from Merck. HPLC-grade solvents, Acetonitrile and methanol, were also purchased from Merck. MethylP (>99%), ButylP (>99%), and EthylP (>99%) analytical standards were also purchased from Merck. Methanol solvent was utilized to prepare a model solution of 1,000 mg/L of MethylP, ButylP, and EthylP. The model solution was stored in the refrigerator at a temperature of 4 °C. Dilutions of the working solutions were prepared using ultra-pure water from the Direct-Q 3UV-R purification system. The parameters of ultrapure water were the resistivity of 18.2 MΩ cm at 25 °C and the electrical conductivity of 0.056 μS/cm. The pH was around 6.6. Prior to analysis, samples were filtered with 0.22 µm polytetrafluoroethylene filters purchased from Separations Scientific SA (Pty) Ltd. Avocado seeds were gathered from the villages surrounding Thohoyandou, Limpopo, South Africa.

### 2.2 Instrumentation

The bonding structures and key functional groups of the synthesized magnetic activated carbon biosorbent were investigated using a PerkinElmer Spectrum 100 FTIR spectrometer (PerkinElmer, United States). The functional groups of the biosorbents were analyzed using Fourier Transform Infrared (FTIR) spectroscopy in the range of 4,000–500 cm^-1^. The elemental composition, nanostructure, and morphology of the biosorbents were determined using scanning electron microscopy (SEM) and energy-dispersive X-ray spectroscopy (EDS) with a TESCAN VEGA 3 XMU instrument (LMH, Czech Republic). An X’Pert XRD (PANalytical BV, Netherlands) equipped with a Cu Ka (k = 0.15406 nm) radiation was used to study the crystallinity of the synthesized magnetic activated carbon. Nitrogen adsorption–desorption isotherms (Autosorb 6200 EPDN XR-AG-AG, Micrometrics Instrument Corporation, Norcross, United States) were utilized to analyze how pore diameters and the specific surface area of the bioadsorbent at a temperature of 77 K. Prior to the analysis, the samples were degassed for 5 h at 300 °C. A sonication bath (Eins-Sci Ultrasonic Cleaner, South Africa) operating at a normal setting of 25 °C was used to perform a dispersing operation on solutions containing analytical components. The sample separation process occurred through a Hermle Z366 centrifuge (Hermle Labortechnik GmbH, Germany) to separate any remaining fine particles from the sample solution, ensuring it is clean and safe for HPLC analysis.

### 2.3 Chromatography conditions

A Shimadzu HPLC (Prominence-i LC-2030C 3D Plus, Japan) equipped with a photodiode array detector was used to analyze the targeted analytes. Chromatographic outputs were recorded at wavelengths of 256 nm for the analytes. A wavelength of 256 nm was chosen to ensure high sensitivity and compatibility with the HPLC mobile phase. The Shimadzu Shim-pack GIST C18 column (150 mm × 4.6 mm, 5 µm) was operated at an oven temperature of 30 °C. The analysis employed gradient elution, which blended water (mobile phase A) and methanol (mobile phase B) in relative percentages programmed as follows: 80:20 (A: B) at 0 min, 60:40 at 1 min, 40:60 at 7 min, and 80:20 at 8 min. The HPLC system was operated at a flow rate of 1.0 mL/min. A Shimadzu Shim-Pack Velox 3.0 mm C18 Guard column (2.7 µm) was used for the analysis.

### 2.4 Sample collection

Inlet and outlet water samples were obtained from the Muledane Wastewater Treatment Plant (WWTP 1) (Thohoyandou, Limpopo, South Africa) and Tzaneen Wastewater Treatment Plant (WWTP 2) (Tzaneen, Limpopo, South Africa). The samples were collected in clean amber glass bottles and stored in a 4 °C refrigerator for up to 1 week before analysis.

### 2.5 Synthesis procedure of activated carbon and magnetic activated carbon

#### 2.5.1 Synthesis of activated carbon from avocado seeds

The synthesis procedure followed the method of Blachnio et al. (Blachnio et al., 2020) with modifications. Avocado seeds were grated into tiny pieces, washed with deionized water, dried overnight at 90 °C, and ground into a fine powder. The three activated carbons were synthesized by impregnating avocado seed powder with 10%, 30%, and 50% KOH solutions separately (1:4) and left for 3 h. The three solutions were then filtered and washed with 0.1 M HCl, followed by deionized water, to remove impurities. Their pH was adjusted to 7.0. The powdered avocado seed, which had been carbonized at 600 °C for 2 h, was then chemically activated at different KOH concentrations (Blachnio et al., 2020; Thuy Luong Thi et al., 2021). The obtained activated carbons were washed with deionized water and dried in an oven. The activated carbons prepared were labeled AC 10% KOH, AC 30% KOH, and AC KOH 50%. The synthesis procedure is summarized in Supplementary Figure S1.

#### 2.5.2 *In-situ* preparation of magnetic activated carbon (MAC) from avocado seeds

The coating of the activated carbon with iron oxide was performed using the method described by Lestari et al. (Lestari et al., 2021) with modifications. The magnetic-activated carbon was prepared by dissolving 4 g of FeSO_4_·7H_2_O and 8 g of FeCl_3_·6H_2_O in 200 mL of deionized water. A hotplate stirrer was used to heat the solution to 90 °C. 4 g of the above-prepared 10%, 30%, and 50% activated carbons were each added to separate portions of the same solution, resulting in three individual mixtures. Subsequently, 5 M (100 mL) of sodium hydroxide was gradually added to each solution and stirred for 10 min. The concoction was then placed in a water bath at 80 °C for 90 min. The formed Magnetic-activated carbon was collected using magnetic separation, washed, and dried at 80 °C for 24 h. The biosorbents prepared were labeled 10% KOH-MAC, 30% KOH-MAC, and 50% KOH-MAC.

### 2.6 Dispersive magnetic solid phase microextraction (DMSPME)

The procedure for DMSPME was conducted as follows: A specific amount of magnetic-activated carbon bioadsorbent was weighed into a vial containing a model solution containing MethylP, EthylP, and ButylP of 100 μg/L. The resulting mixture was subjected to sonication using an ultrasound water bath for 10–30 min. The supernatant was separated from the magnetic adsorbent (with the extracted analytes) using an external magnet. The bioadsorbent was retained, while the supernatant was decanted. Then, the analytes of interest were eluted through sonication using methanol. The analysis of the analyte concentrations was performed using HPLC-PDA. This analysis was conducted in replicates.

### 2.7 Optimization strategy

#### 2.7.1 Univariate optimization

A suitable bioadsorbent composition and eluent solvent were determined through a univariate approach. This method aimed to identify which composition of the KOH of the bioadsorbent and eluent solvent achieved the highest recoveries for extracting the three parabens from water. Three various compositions of KOH (10%, 30%, and 50%) for chemical activation were tested. During the testing of the bioadsorbent composition process, the following experimental conditions were fixed: 6.5 pH of the sample; 5 mL sample volume; 22.5 mg of the biosorbent mass; 30 min extraction time; and 850 µL eluent volume (acetonitrile). It was only the biosorbent that was varied. The best eluent solvent was selected in a similar manner. The best bioadsorbent (30% KOH-MAC), with an 850 µL volume eluent, a 6.5 pH sample, 22.5 mg of bioadsorbent mass, and an extraction time of 30 min, was kept constant, and only the eluent solvent was varied. The tests were conducted using acetonitrile, methanol, and a 50:50 (v/v) mixture of acetonitrile and methanol.

#### 2.7.2 Multivariate optimization of dispersive magnetic solid phase microextraction (DMSPME)

A multivariate strategy was utilized to enhance the dispersive magnetic solid-phase extraction method. Significant factors were identified during the initial screening phase through a fractional factorial design (FFD). These identified factors were then further optimized using a central composite design (CCD) to establish the optimal conditions for the preconcentration and simultaneous extraction of trace amounts of parabens in water. The parameters determined included pH of the sample, mass of adsorbent (MA), extraction time (ET), and eluent volume (EV). The selected numerical ranges for the parameter optimizations are outlined in Table 1.

**TABLE 1:** Variables that are independent and their corresponding levels in the design of experiments.

Parameters	Minimum	Central	Maximum
Eluent volume (µL)	800	900	1,000
Extraction time (min)	15	22.5	30
pH	4	6.5	9
Mass of adsorbent (mg)	20	22.5	30

### 2.8 Point of zero charge of the 30%KOH-MAC

Understanding the PZC of the magnetic activated carbon (MAC) is vital for analyzing its adsorption characteristics (Abdulkadir et al., 2025). To determine the PZC of the 30%KOH- MAC biosorbent, a method from the literature was followed (Zeng et al., 2021). Various 30 mL pH solutions were prepared in conical flasks. The pH levels were modified from 2 to 10 by using ammonium hydroxide and acetic acid. Each flask received 22.5 mg of 30% KOH-MAC and was sonicated for 48 h. After this, the final pH of each solution was recorded. The PZC was determined from a plot of (final pH–initial pH) versus initial pH.

### 2.9 Method validation

The DMSPME method was validated by assessing the linear range, limit of detection (LOD), limit of quantification (LOQ), and both intraday and interday precision. This validation was conducted based on a study conducted by (Selahle et al., 2022). For the intraday precision, a solution with 100 μg/L of each analyte (MethylP, ButylP, and EthylP) was analyzed in ten separate trials (n = 10). The analysis was conducted over 5 days (n = 5) to assess interday precision and was conducted in replicates. Additionally, a spike recovery approach was used to test the method’s trueness. Three levels (10, 50, and 100 μg/L) were added to the inlet and the outlet wastewater samples, and each sample was analyzed three times. The percentage recoveries of spiked samples were determined using the Formula 1,

$$\%R=\frac{\left[\text{Found}\right]-\left[0\right]}{\left[\text{added}\right]}$$ (1)

In this formula, [Found] indicates the concentration measured in the spiked sample, [0] is the concentration of the blank sample (unspiked sample), and [added] is the concentration introduced for spiking. To evaluate the linearity of the method, a blank inlet and outlet wastewater sample were spiked to create seven standard solutions with concentrations from LOQ to 35 μg/L for each analyte, and this was conducted in triplicate. The DMSPME method was used to extract and preconcentrate these standards, facilitating the creation of a seven-point calibration curve. The LOD and LOQ were calculated using Equations 2, 3, respectively:

$$LOD=\frac{3\text{xSD}}{m}$$ (2)

and

$$\text{LOQ}=\frac{10\text{xSD}}{m}$$ (3)

In these equations, *SD* refers to the standard deviation of the peak areas from 3 to 10 replicates of the minimum concentration in the linear range, while *m* denotes the slope of each calibration plot for the target analytes. The enrichment factor (EF) was calculated as the ratio of the final concentration of analytes after extraction to the initial concentration of analytes before extraction.

### 2.10 Adsorption capacities

Under optimal conditions, the biosorbent’s adsorption capacity towards the parabens was investigated. 22.5 mg of the biosorbent was combined with 30 mL of a model solution that contained 100 mg/L of each paraben, and the pH was adjusted to 6.5. After that, the mixture was ultrasonically agitated for 32.5 min. The quantity of each paraben adsorbed onto the biosorbent surface was calculated using Equation 4. This analysis was conducted in replicates.

$$Qe=\frac{\left(\text{Ci}-\text{Ce}\right)}{M}V$$ (4)

where Qe (mg/g) signifies the quantity of MethylP, EthylP, and ButylP that is adsorbed per gram of the adsorbent. Ci and Ce (mg/L) represent the initial and equilibrium concentrations of the solution, respectively. L stands for the volume of the aqueous solution, and M (g) indicates the mass of the adsorbent utilized.

### 2.11 Adsorption kinetics

Adsorption kinetics are determined to understand the rate-limiting steps and the adsorption mechanisms for MethylP and EthylP using 30% KOH-MAC. The reaction kinetics models of pseudo-first-order (PFO) and pseudo-second-order (PSO), as well as intraparticle diffusion, were employed to study the adsorption kinetics. These equations are clearly explained in Supplementary Table S2. The data for adsorption kinetics studies were obtained using a sample volume of 5 mL, a mass of adsorbent of 20 mg, an initial concentration of 10 mg/L, a sample pH of 6.5, and an interaction time varied from 10 to 35 min. The table below presents the equations used to estimate the parameters for the kinetic models.

### 2.12 Adsorption isotherms

Langmuir, Freundlich, Sips, and the Dubinin-Radushkevich (D-R) adsorption isotherm models were used to understand the relationship and interactions between the parabens adsorbed on the surface of 30% KOH-MAC. The isothermal equations are further explained in Supplementary Table S3. The data for adsorption isotherm studies were obtained using a sample volume of 5 mL, a mass of adsorbent of 20 mg, an interaction time of 30 min, a sample pH of 6.5, and an initial concentration ranging from 5 ppm to 50 ppm.

### 2.13 Reusability studies through extraction cycles

Studies were conducted on the reusability and regeneration through a series of adsorption-desorption experiments. 22.5 mg of the bioadsorbent mass was measured and added to a sample bottle. Then, 5 mL of the model solution containing MethylP, EthylP, and ButylP was added to the sample bottle. The mixture underwent sonication for 32.5 min before being eluted with 875 μL of methanol. The eluent, which contained targeted parabens, was analyzed using HPLC-PDA. This was conducted several times using a similar bioadsorbent lost its functionality to adsorb high percentages of the parabens. The method and the data obtained were used to conduct the statistical analysis.

## 3 Results and discussion

### 3.1 Characterization

#### 3.1.1 Fourier-transform infrared spectroscopy (FTIR)

**FIGURE 1:**
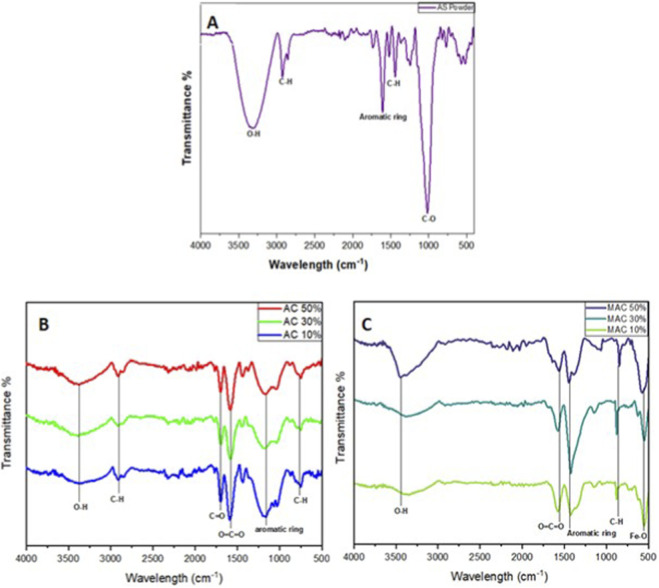
FTIR spectra illustrating the functional groups present in **(A)** Avocado seed powder, **(B)** Activated carbon with 10%KOH, 30%KOH and 50%KOH, **(C)** Magnetic activated carbon with 10%KOH, 30%KOH and 50%KOH.

FTIR spectra were obtained to assess the functional groups of the synthesized biosorbent. Figures 1A–C indicate that the raw avocado seed powder and different percentages of KOH are used in the activation and magnetization of the synthesized bioadsorbent. According to Figure 1, the FTIR spectra showed few significant differences between the AC and MAC. However, it can be seen that the broadness of the OH peak increased after the introduction of the KOH. The sharp peak of C-O observed in Figure 1A indicated that the carbon structure was bare, not having anything attached to it. The last peak at 500 cm^-1^ also indicates that it is intensified after the addition of iron. The three samples exhibited nearly identical sets of vibrational FTIR peaks. A broad peak at around 3,500 cm^−1^ was observed, suggesting that the O-H stretching vibrations are from the intermolecular hydrogen bonding. These OH peaks were broad and wide, indicating chemical activation. Another peak was also observed at 2,905 cm^-1^ due to the C–H stretching. Other peaks were observed at 1701 cm^-1^ and 1,586 cm^-1^, which were assigned to the C=O stretching of carboxylic acids and the O=C=O stretching of carboxylates, respectively. This was followed by a peak at 1,439 cm^-1^, corresponding to the aromatic ring. The peak at 1,179 cm^-1^ was attributed to the C–O stretching of phenols. Subsequently, a peak at 752 cm^-1^ was assigned to C–H of the aromatic rings. Figure 1B indicated an additional band at 689–500 cm^-1^. This was due to Fe-O, which was added during the magnetization stage. This peak was expected as it confirms the successful formation of the magnetic activated carbon.

#### 3.1.2 Scanning electron microscopy (SEM)

**FIGURE 2:**
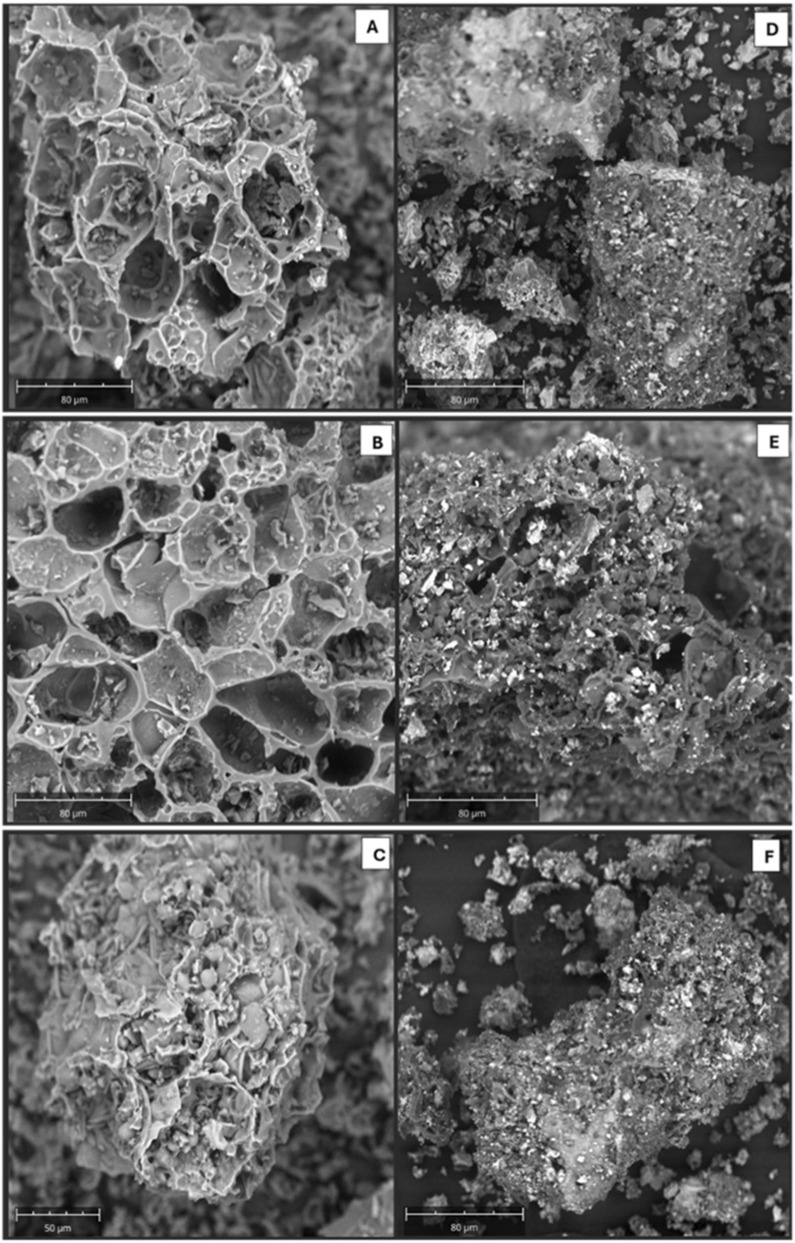
SEM images highlighting the surface morphology of **(A)** 10% AC, **(B)** 30% AC, **(C)** 50% AC, **(D)** 10%KOH-MAC, **(E)** 30%KOH-MAC, and **(F)** 50%KOH-MAC.

SEM of the AC (Figures 2A,B) reveals high porosity with a well-defined, interconnected pore network. The smooth pore walls suggest a lightweight structure, potentially beneficial for adsorption. Figure 2C did not indicate well-defined pores. After the introduction of iron particles, the pores of the AC became smaller. This is illustrated in Figures 2D–F. However, some of the pores were still visible in Figure 2E. This proved that the incorporation of the Fe_3_O_4_ into the AC was successful. However, literature has indicated that when iron fills in the pores of AC, it reduces the surface area of the sorbent formed and might affect the adsorption process (Montaño et al., 2024). Nonetheless, the incorporation of iron strengthens the AC, and the magnetization facilitates the easy recovery of the biosorbent using an external magnet, making it more stable in an aqueous environment. Using an external facilitates easy recovery of the biosorbent. This is also crucial for preventing degradation of the biosorbent over time.

#### 3.1.3 Energy dispersive X-ray spectrometer (EDS)

**FIGURE 3:**
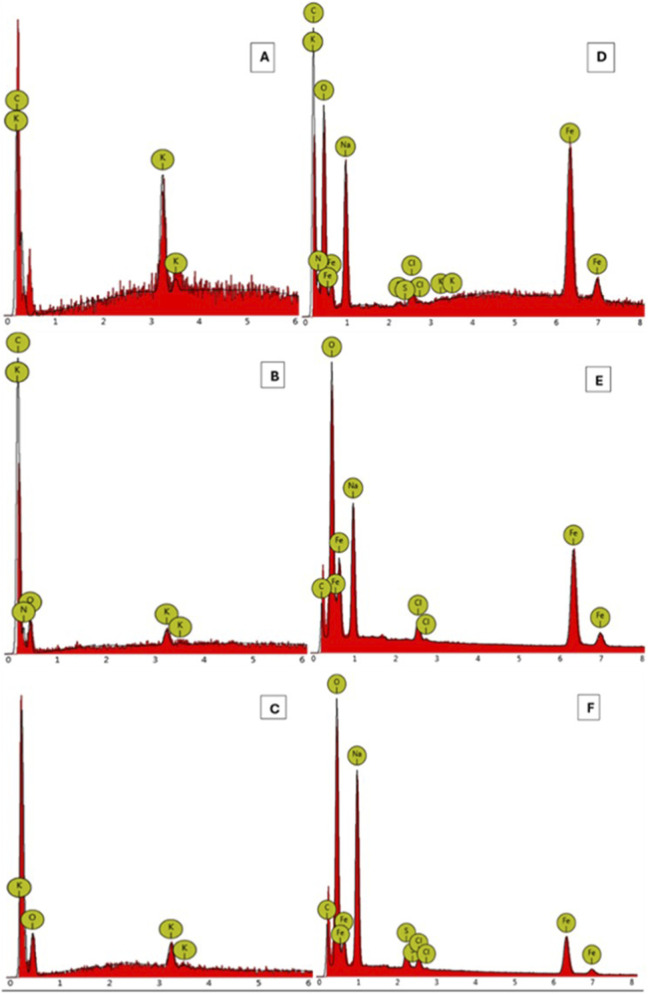
EDS spectra of **(A)** 10%KOH-Activated Carbon (AC), **(B)** 30%KOH-Activated Carbon, **(C)** 50% KOH-Activated Carbon, and **(D)** 10%KOH-Magnetic Activated Carbon, **(E)** 30%KOH-Magnetic Activated Carbon, and **(F)** 50%KOH-Magnetic Activated Carbon.

The EDS spectra in Figures 3A–C revealed the presence of all the expected elements: carbon, oxygen, and potassium. Nitrogen was also observed in Figure 3B, which was considered an impurity. This was expected because avocado seeds contain many other elements. Figures 3D–F EDS spectrum revealed the presence of iron, carbon, oxygen, potassium, chlorine, and sodium. All these elements were expected except chlorine and sodium. Cl and Na were present in the ferric chloride and sodium chloride salts used in the synthesis. This indicated that the synthesized MAC contained some impurities. The peak of Fe in Figures 3D–F indicated that the incorporation of Fe_3_O_4_ into the activated carbon was successful, indicating that the desired biosorbent was successfully synthesized.

#### 3.1.4 X-ray diffraction (XRD)

**FIGURE 4:**
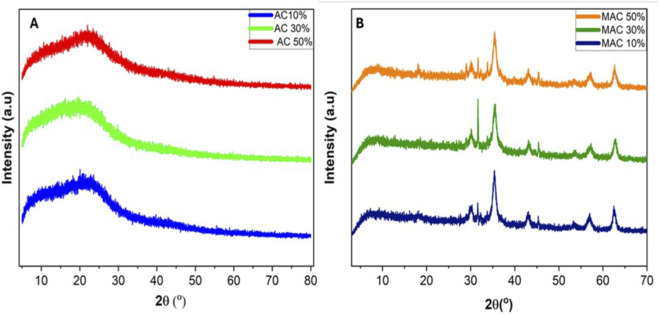
XRD spectra of **(A)** 10%KOH-Activated Carbon, 30%KOH-Activated Carbon, 50%KOH-Activated Carbon, and **(B)** 10%KOH-Magnetic Activated Carbon, 30%KOH-Magnetic Activated Carbon, and 50%KOH-Magnetic Activated Carbon.

XRD spectra were obtained to evaluate the crystallinity of the synthesized biosorbent. Figures 4A,B indicate the XRD spectrum for the avocado seeds activated and magnetic activated carbon. Figure 4A indicates that AC was amorphous. These findings aligned closely with a study reported by (Lestari et al., 2021). Figure 4B indicates that introducing Fe_3_O_4_ to AC transformed the amorphous structure to a crystalline structure. Additional distinct peaks were observed with 2-Theta values at approximately 32.1°, 37.4°, 45.1°, 55.4°, 56.9°, and 62.5°. These peaks indicated the presence of Fe_3_O_4_ particles in the AC, which formed MAC. They also correspond to the JCPDF of iron oxide XRD spectra.

#### 3.1.5 Vibrating sample magnetometry (VSM)

The magnetic property of 30% KOH-MAC was studied using vibrating sample magnetometry. The findings are presented in Supplementary Figure S2 as a plot. Based on the plot, the saturation magnetization value of 30% MAC was around 10.48 emu/g. The magnetic property of 30% activated MAC is significantly lower in comparison to other magnetic materials reported in the literature. The 30% KOH-MAC decrease in magnetic property indicated the successful incorporation of Fe_3_O_4_ particles into AC. 30% KOH-MAC has activated carbon embedded in it, hence its low magnetic property. Even so, the magnetization value of 30%KOH-MAC was sufficient for magnetic separation using an external magnetic field during the extraction process.

#### 3.1.6 Brunauer–emmett–teller (BET)

**FIGURE 5:**
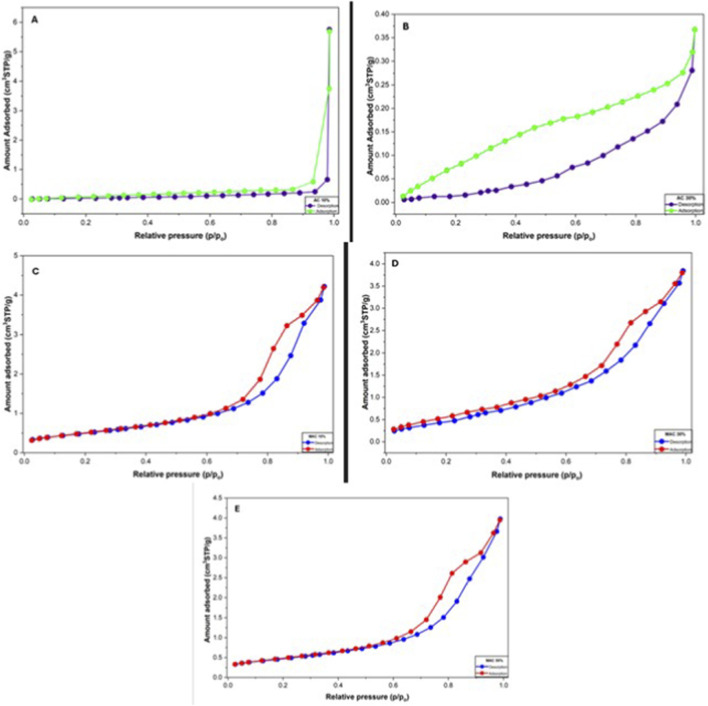
BET adsorption and desorption isotherm graphs for **(A)** 10% AC, **(B)** 30% AC, **(C)** 10% KOH-MAC, **(D)** 30% KOH-MAC, and **(E)** 50% KOH-MAC, illustrating surface area and porosity characteristics.

The Nitrogen adsorption–desorption isotherm analysis of the MACs (10% KOH, 30% KOH, and 50% KOH) was performed to evaluate the change in surface area and to determine the porous nature of the biosorbents. This was done using nitrogen adsorption–desorption isotherms. From Table 2, the surface area of the AC 30%KOH exhibited the highest value at 51 m^2^/g, compared to the AC 10% KOH, which had a surface area of 43 m^2^/g. After magnetization, there was a decrease in surface area; however, the BET surface area of the 30% KOH-MAC biosorbent remained the highest at 30.156 m^2^/g, making it the most suitable option among the 10% KOH-MAC and 50% KOH-MAC biosorbents. A similar trend was observed in terms of pore volume, with 30% KOH-MAC exhibiting a pore volume of 0.075 cm^3^/g, indicating greater pore structure development. The average pore diameters for all the MACs ranged from 2.33 to 2.36 nm, which positions them at the boundary between microporous and lower mesoporous classification (Colomer, 2024). 30% KOH-MAC resulted in good porosity and surface accessibility, which can enhance adsorption performance. Figures 5A–E showcases the adsorption-desorption behavior of the synthesized ACs and MACs. In Figures 5A,B, we can see the nitrogen adsorption-desorption isotherms for AC 10% and AC 30%. Both of these plots reveal a type IV isotherm accompanied by noticeable hysteresis loops, Figure 5A shows an increase around 1.0 P/P_0_, which suggests the presence of mesopores. On the other hand, Figure 5B displays a more pronounced hysteresis loop, indicating that it has enhanced mesoporosity. Figures 5C–E also illustrate type IV isotherms with H3-type hysteresis loops, which further confirm that MACs have mesoporous characteristics. When comparing the MACs and ACs, there is a slight decrease in nitrogen adsorption. This decline indicates a reduction in both surface area and pore volume following the magnetization process. It appears that the magnetic nanoparticles, which are deposited during magnetization, filled some of the internal pore spaces or covered the surface of the activated carbon.

**TABLE 2:** Surface area, pore volume, and average pore diameter of 10%KOH-MAC, 30%KOH-MAC and 50%KOH-MAC derived from N_2_ adsorption–desorption isotherms.

BET parameters	AC 10%	AC 30%	10%KOH-MAC	30%KOH-MAC	50%KOH-MAC
BET surface area (m^2^/g)	43	51	28	30	23
Pore volume (cm^3^/g)	0.2	0.2	0.1	0.1	0.1
Pore diameter (nm)	2.6	2.5	2.3	2.1	2.4

#### 3.1.7 Point of zero charge of 30% KOH-MAC

The pH point of zero charge (pHpzc) of the 30% KOH-MAC was determined to understand the surface charge characteristics of the biosorbent to help predict the interaction between the 30% KOH-MAC 30% and the parabens (Kosmulski, 2023). The 30% KOH-MAC pHpzc is presented in Supplementary Figure S3, which was found to be 7.6, indicating that its surface has a positive charge at pH values below 7.6. The surface of the 30% KOH-MAC was positively charged (proton-rich), which is good for the electrostatic interaction of negatively charged species such as electron-rich parabens (R–COO^-^). Furthermore, the surface had negative charges at pH values above 7.6, potentially resulting in electrostatic repulsion between the 30% KOH-MAC and the parabens.

### 3.2 Optimization strategy

#### 3.2.1 *Univariate optimization* (investigating the percentage of KOH used for activation, and selection of eluent solvent)

**FIGURE 6:**
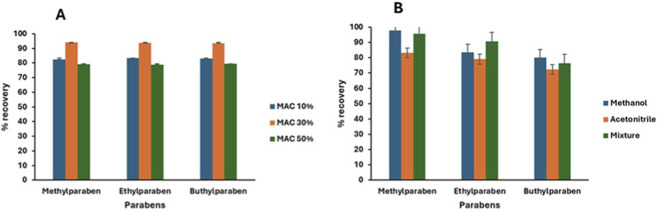
Illustration of the selection of **(A)** the best activation percentage of the biosorbent and **(B)** the best eluent solvent for preconcentration and extraction of parabens.

Choosing the most effective biosorbent for extracting and preconcentrating parabens from water bodies was achieved by evaluating the effectiveness of different MACs at varying KOH concentrations (10%, 30%, and 50%). From the percentage recoveries shown in Figure 6A, 30% KOH- MAC had better percentage recoveries of the parabens than 10% and 50%. During the magnetization stage, Fe_3_O_4_ occupied the pores of AC, leaving some pores available for utilization during the adsorption process. This was also supported by an SEM image (Figure 2E), which indicated that after magnetization with Fe_3_O_4_, the 30% KOH-MAC still showed more pores than the 10% and 50% samples. Additionally, the BET analysis (Table 2) indicated that 30% KOH-MAC had a surface area and pore volume higher than activated 10% and 50% MAC. This meant that 30% KOH-MAC would adsorb parabens better than the other two materials, being activated at 10% and 50%.

To achieve a high recovery percentage of parabens and maximize the extraction of parabens from the biosorbent surface, the appropriate eluting solvent was selected by evaluating the effectiveness of various solvents. Using triplicate adsorption extraction studies, the extraction capabilities of various organic solvents (MeOH, ACN, and a 50:50 (v/v) MeOH: ACN mixture) were evaluated. The polarity of methanol and parabens allowed for dipole-dipole interactions and hydrogen bonding to occur between the solvent and the adsorbate, promoting the effective dissolution of parabens in methanol and enhancing their adsorption. This implied that the parabens had a high methanol solubility, which improved the compound’s separation and retention. This was supported by Figure 6B, which shows that the plots indicate a high percentage of paraben recoveries when eluted with a methanol solvent. Therefore, methanol was selected as the optimal solvent and used for the subsequent extraction process.

#### 3.2.2 Multivariate optimization

**FIGURE 7:**
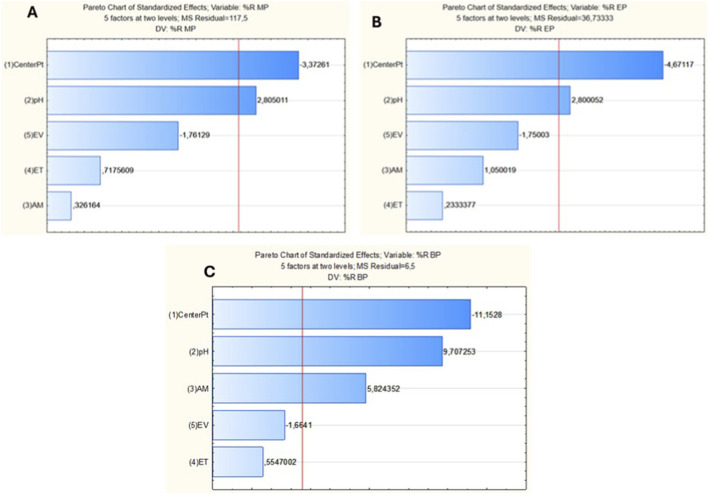
Pareto charts of experimental variables on the preconcentration and simultaneous extraction of **(A)** MethylP, **(B)** EthylP, and **(C)** ButylP; where MA = Mass of adsorbent; ET = Extraction time; and EV = Eluent volume.

The effect of the independent variables: adsorbent sample pH, adsorbate mass (AM), elution volume (EV), and extraction time (ET), on the extraction of parabens, was assessed using a fractional factorial design (FFD) screening process. The optimized experimental conditions are illustrated in Table 1 of the experimental section. The findings were examined and displayed in a Pareto chart utilizing analysis of variance (ANOVA), as revealed in Figure 7. It is depicted that the blue bars for pH crossed the 95% confidence level (red line) from the target analytes’ Pareto charts, suggesting that pH is the most significant factor for the extraction and preconcentration of the three parabens. The other significant factor was adsorbent mass (MA), as it also exceeded the red line, according to Figure 7C. EV was also considered significant, as its blue bar was near the red line. This can also be justified by the fact that methanol had a superior performance in elution. This may be attributed to its enhanced interaction with the sorbed analyte and superior infiltration of the biosorbent microporous network compared with acetonitrile. The ANOVA results in Supplementary Table S4 indicate that the sample pH and eluent volume significantly affect the recoveries of EthylP, MethylP, and ButylP at a 95% confidence level. This is because p-values were greater than 0.05. In addition, high F- and low p-values suggest that the Pareto models provide a significant fit to the described analytical response (%R) as a function of the studied factors.

**FIGURE 8:**
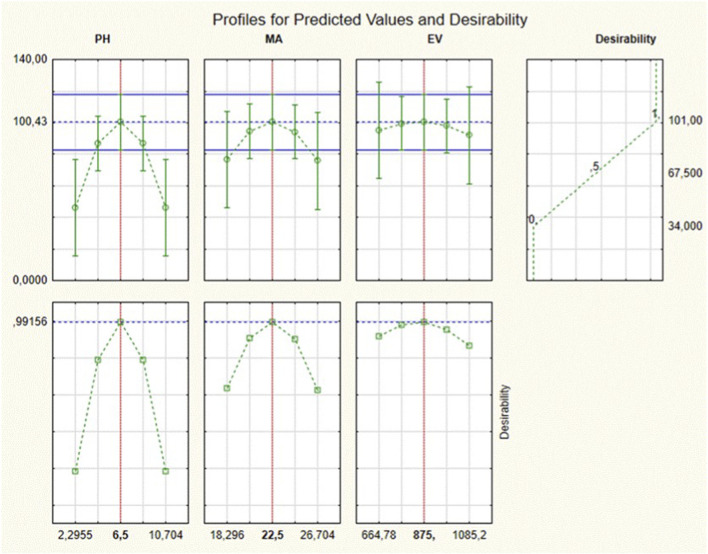
Optimization of independent parameters for targeted parabens using a desirability function.

The desirability functions were utilized for the determination of the precise numerical values of the parameters that are essential to maximizing the target compounds’ extraction recovery (Bayuo et al., 2024). The extraction conditions that afford satisfactory percentage recoveries of the parabens were determined, as shown in Figure 8: pH of 6.5, an adsorbate mass of 22.5 mg, and an eluent volume of 875 µL. These parameters led to satisfactory extraction recoveries of parabens. Therefore, the desirability function successfully optimized the experimental conditions, and further analysis was conducted using these optimized conditions. Multivariate optimization was designed to simultaneously evaluate and optimize multiple experimental variables affecting the efficiency of the analytical procedure. Unlike univariate approaches, which assess one factor at a time, multivariate methods enable the identification of interactions between variables, resulting in a more robust and efficient optimization process.

### 3.3 Method validation

The evaluation of the analytical measurements of effectiveness for the DMSPME-HPLC-PDA method was conducted. This was conducted under the optimum experimental conditions as discussed in the experimental section. As depicted in Table 3, a linear dynamic range (LDR) of LOQ -35 was achieved. The reproducibility and repeatability of the extraction method were also assessed and presented as relative standard deviations (% RSD). Lower intraday and interday were obtained to be in the range of 2.80%–4.50% and 3.40%–4.20%, respectively. These results showed good precision as the RSDs were all below 5%. LODs and LOQs of the method were 0.12–0.19 μg/L and 0.25–0.90 μg/L, respectively. These obtained values conveyed that the DMSPME-HPLC-PDA method was sensitive. Therefore, conducting the analytical performance illustrated that the DMSPME-HPLC-PDA method was effective and sensitive enough for the simultaneous extraction of MethylP, ButylP, and EthylP in wastewater samples. Additionally, the parabens demonstrated comparable enrichment factors, ranging from 41 to 43, confirming the strong preconcentration capability and reliability of the DMSPME-HPLC-PDA method for determining EthylP, MethylP, and ButylP.

**TABLE 3:** Summary of the analytical performance parameters of the DMDMSPE–HPLC–PDA method.

Parameters	EthylP	MethylP	ButylP
Linear range (µg/L)	0.1–35	0.1–35	0.1–35
R^2^	0.95	0.98	0.96
LOD (µg/L)	0.12	0.19	0.17
LOQ (µg/L)	0.90	0.25	0.67
Intraday (%RSD), n = 10)	5	3	3
Interday (%RSD, n= 3 days)	4	4	3
EF	42	43	44

The validity of the presented method was evaluated through analysis of spiked water samples. Inlet and outlet samples from both wastewaters were spiked using three concentrations: 10, 50, and 100 μg/L. The analysis of these concentrations was conducted in triplicate. As shown in Table 4, the recovery percentage (% R) obtained ranged from 99.1% to 101%. The recovery results showed that the matrix effects did not affect the performance of the extraction method. This also indicated that the DMSPME-HPLC-PDA method was appropriate for the preconcentration and simultaneous extraction of the parabens.

**TABLE 4:** Spiking recovery results for influent and effluent wastewater samples containing parabens using the optimized method.

Paraben	Added (µg/L)	Measured value (µg/L) N = 3	%Recovery	%RSD
Methyl	10	9.87 ± 0.1	98.7	1.2
​	50	49.6 ± 1.2	99.2	2.5
​	100	99.8 ± 0.1	99.8	1.3
Ethyl	10	9.91 ± 0.9	99.1	3.0
​	50	50.5 ± 1.7	101	2.1
​	100	99.8 ± 0.1	99.8	3.6
Butyl	10	9.76 ± 0.9	97.6	3.3
​	50	49.8 ± 0.1	99.6	3.2
​	100	99.7 ± 1.4	99.7	1.7

### 3.4 Application of the developed method

According to Supplementary Table S5, MethylP, EthylP, and ButylP were found in both wastewater treatment plants of interest. The concentrations found in the inlets for WWTP 1 and WWTP 2 ranged from 6.10 to 17.39 μg/L. Higher concentrations were anticipated in the inlet samples, as the water had not undergone any treatment. The chromatograms for the analysis are shown in Supplementary Figure S6. The outlet ranged from <LOQ to 10.39 μg/L. These concentrations were found to be alarming when compared to the Acceptable Daily Intake (ADI) for consumable goods (Al-Qudaihi et al., 2025). These concentrations indicated a potential health risk, and it was alarming for Butyl since it was mentioned to be unsafe for consumption (Bernauer et al., 2023). This suggests that WWTPs are not sufficiently equipped to effectively remove all types of organic contaminants, including parabens. Additionally, this also indicated the efficiency of the DMDMSPE-HPLC-PDA method for the simultaneous extraction of parabens.

### 3.5 Adsorption capacities and possible interaction mechanisms

The capability of the prepared 30% KOH-MAC from avocado seeds to adsorb the three targeted parabens was examined. This was conducted by examining the adsorption capacities. The determined adsorption capacities of the 30%KOH- MAC were found to be 255.3, 266.4, and 264.0 mg/g for EthylP, ButylP, and MethylP, respectively. These calculations were obtained from adsorption studies. These adsorption capacities are similar to other reported adsorption capacities utilizing activated carbon adsorbents (Moreno-Marenco et al., 2019) and (Mashile et al., 2018). This indicated that the prepared 30% KOH-MAC biosorbent has the same efficacy as the other adsorbents; however, the biosorbent is more advantageous since it is both affordable and environmentally friendly.

**FIGURE 9:**
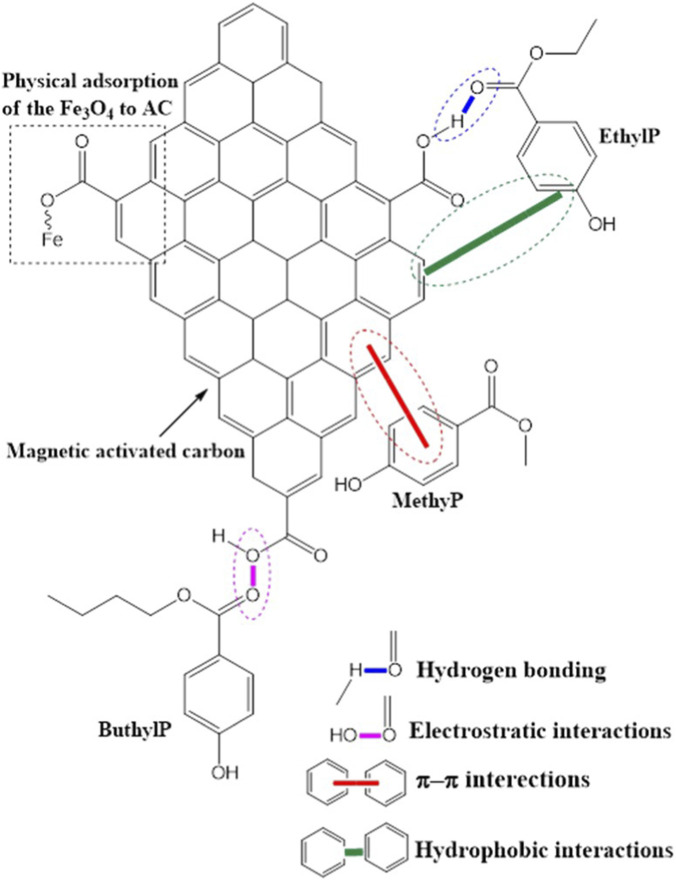
Schematic presentation of the MethylP, EthylP, and ButylP adsorption mechanism onto the surface of 30% KOH-MAC biosorbent in an aqueous environment.

The possible interaction mechanisms between the prepared 30% KOH-MAC biosorbent and the targeted parabens were investigated. The hydrophobic interactions were the first possible interaction mechanism between the MAC surface and the alkyl and aromatic ring of the parabens; this behavior is shown by the green bond in Figure 9. This was due by the optimum value of pH 6.5 being close to the pHpzc 7.6 of the MAC (Supplementary Figure S3), the surface tends to be more hydrophobic because the ionization of surface groups is minimal, and therefore enhances the extraction of the parabens (Monroe et al., 2020). The electrostatic interactions were the second possible interaction mechanism; this behavior is illustrated by the purple bond in Figure 9. This occurs between the surface of the 30% KOH-MAC charges, which are positive, and the surface of the analytes’ charges, which are negative or vice versa (Osman et al., 2023). The electrostatic interaction mechanism depends on the pH of the sample. When the pH is lower than the pKa of the analytes, they exist in a neutral state. In divergence, when the pH exceeds the pKa, the parabens become negatively charged relative to the pHpzc of the adsorbent, which governs the surface charge. The optimum pH from optimization was 6.5, while the parabens pKa value ranged between 8.2 and 8.9, and the pHpzc of the 30% MAC was 7.6. Moreover, the third possible mechanism was the hydrogen bonding between MAC’s hydrogen bond acceptors and parabens’ hydrogen bond donors. This bond is illustrated in Figure 9 by the blue line. Lastly, another possible mechanism was the π-π interaction between the aromatic rings of the 30% KOH-MAC and the parabens. This bond is indicated by the red bond in Figure 9.

### 3.6 Adsorption kinetics

To further elucidate the adsorption mechanism, adsorption studies were conducted, and the data were fitted to linearized equations for pseudo-first-order and pseudo-second-order reaction kinetics. The linear plots and the respective constant parameters are depicted in Supplementary Figure S4 and Table 5, respectively. Based on the constant values and the statistical parameters shown in Table 5, the adsorption data for the studied parabens fitted better to the Pseudo-first-order model. This was validated by the high R^2^ values along with the low χ^2^, SEE, RSS, and RMSE values. These findings suggest that the uptake mechanisms of parabens by the 30% KOH-MAC are associated with a physisorption process, which involves electrostatic interactions and weak van der Waals forces.

**TABLE 5:** adsorption kinetics statistics values of the parabens onto the surface of the 30%KOH-MAC.

Pseudo-first-order			Pseudo-second-order		
Parameters	EthylP	MethylP	Parameters	EthylP	MethylP
$R^{2}$	0.92	0.90	$R^{2}$	0.73	0.65
${\text{Adj}.R}^{2}$	0.90	0.87	${\text{Adj}.R}^{2}$	0.66	0.56
$X^{2}$	0.06	0.05	$X^{2}$	0.74	1.3
SEE	0.34	0.32	SEE	0.52	0.58
RSME	0.28	0.27	RSME	0.43	0.47
RSS	0.46	0.43	RSS	1.09	1.35

#### 3.6.1 Intra-particle diffusion

The solute transfer process in solid-liquid sorption is typically characterized by three main stages: external mass transfer, intraparticle diffusion, and adsorption onto the active binding sites (Munyegaju, 2022). The intraparticle diffusion plots for the uptake of parabens by 30% KOH-MAC are illustrated in Supplementary Figure S5 and summarized in Table 6. These findings indicate that the adsorption process exhibits a multi-linear behavior, suggesting that the uptake of parabens by 30% KOH-MAC is not only controlled by intra-particle diffusion (Morales et al., 2025). The plots for the parabens in Figure 11 revealed two linear regions that deviate from the origin. The first region represents the boundary layer diffusion with a high $K_{\text{id}}$ , indicating a rapid uptake of the parabens within the initial minutes, likely due to electrostatic interactions and hydrogen bonding. This is followed by intra-particle diffusion, characterized by a low $K_{\text{id}}$ , which suggests a slower diffusion of the parabens into 30%KOH-MAC. The positive C intercept related to boundary layer thickness indicates that intraparticle diffusion is not the only rate-limiting step in the process.

**TABLE 6:** Intra-particle diffusion kinetics statistics values of the parabens onto the surface of the 30%KOH-MAC.

EthylP			MethylP		
Parameters	Stage 1	Stage 2	Parameters	Stage 1	Stage 2
$R^{2}$	0.76	0.99	$R^{2}$	0.768	1
$K_{\text{id}}$	0.706	0.109	$K_{\text{id}}$	0706	0.104
C	−2,166	1.428	C	−2.166	1.454

### 3.7 Adsorption isotherms

**FIGURE 10:**
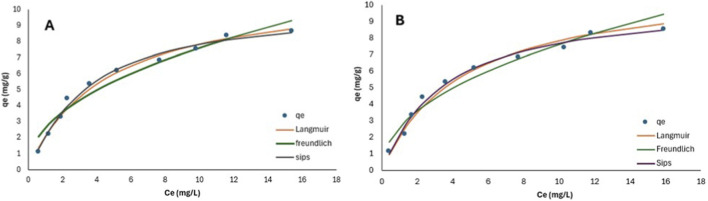
**(A)** MethylP non-linear plots according to the Langmuir, Freundlich, and Sips isotherm models; **(B)** EthylP non-linear plots according to the Langmuir, Freundlich and Sips isotherm models.

Figure 10 and Table 7 depict the nonlinear plots for the Langmuir and the Freundlich isotherm and their parameters, respectively. Based on the values of the root mean square error (RMSE), residual sum of squares (RSS), and chi-squared (χ^2^) (Table 7), the adsorption process for both EthylP and MethylP followed the Langmuir adsorption isotherm model. Therefore, this suggests a homogeneous adsorption phenomenon. While the values of the coefficient of determination (R^2^) and the adjusted correlation coefficient (adjusted R^2^) for the Freundlich model are closer to 1, they reflect the strength of the relationship between the experimental data and the predicted data. However, these values do not account for the magnitude of the residuals. The Sips isotherm equilibrium data fitted well with R^2^ = 0.98 for the parabens, and the Sips heterogeneity factor for MethylP and EthylP was 1.05 and 1.15, respectively, indicating that the 30% KOH-MAC surface was homogeneous, with adsorption occurring predominantly as a monolayer. In line with the findings of the Langmuir model, the small departure from unity points to a limited number of higher-energy adsorption sites.

**TABLE 7:** Adsorption isotherm statistics values of the parabens onto the surface of the 30%KOH-MAC.

Langmuir			Freundlich		
Parameters	MethylP	EthylP	Parameters	MethyP	EthylP
$Q_{m}$	11.37	11.37	$K_{f}$	2.613	2.613
$K_{L}$	0.22	0.22	n	2.15	2.15
$R^{2}$	0.87	0.89	$R^{2}$	0.95	0.95
${\text{Adj}.R}^{2}$	0.86	0.88	${\text{Adj}.R}^{2}$	0.94	0.94
$X^{2}$	0,29	0.12	$X^{2}$	0.85	1.3
SEE	0.38	0.31	SEE	0.60	0.60
RSME	0.34	0.27	RSME	0.53	0.54
RSS	1.12	0.78	RSS	2.89	2.91

Sips					
Parameters	​	MethylP	​	EthylP	​
$R^{2}$	​	0.97	​	0.98	​
$Q_{m}$	​	10.16	​	10.00	​
$K_{s}$	​	0.29	​	0.30	​
N	​	1.05	​	1.14	​

#### 3.7.1 Dubinin Radushvich (D-R)

**FIGURE 11:**
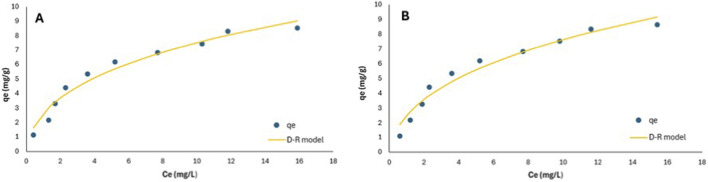
**(A)** EthylP non-linear plot according to the Dubinin Radushvich (D-R) isotherm model; **(B)** MethylP non-linear plot according to the Dubinin Radushvich (D-R) isotherm model.

The D-R model was also plotted as show in Figure 11 and Table 8 to determine the effect of the porous nature of the composite as well as to determine if it was physisorption or chemisorption, using the mean free energy (E) of the adsorption process, which is defined as the free energy change when 1 mol of adsorbate is transferred to the surface of the solid from infinity in solution. Therefore, if the mean free energy is between 8 and 16 kJ/mol, the process is due to chemisorption, and if the mean free energy is lower than 8 kJ/mol, it is physisorption. Table 8 shows the parameter model of the parabens adsorption onto the 30% KOH-MAC. The obtained E value was found to be between 8 and 16 kJ/mol; thus, the process occurred through a chemisorption process with the surface and composition of the boisorbent.

**TABLE 8:** Dubinin Radushvich values of the parabens onto the surface of the 30%KOH-MAC.

Parameters	MethylP	EthylP
$R^{2}$	0.97	0.94
$Q_{m}$	0.31	0.32
Β	3.38 × 10^−3^	3.55 × 10^−3^
E	12.17	11.86

### 3.8 Comparison of the DMSPME-HPLC-PDA technique with previous studies

The DMSPME-HPLC-PDA method was compared to other reported techniques for the simultaneous extraction of parabens from water samples. This comparison was made based on the type of sorbent, the analytical instruments used for quantification, and certain analytical figures such as LOD and LOQ, as well as similar applications. The comparison is presented in Supplementary Table S6. The presented DMSPME-HPLC-PDA method had an LOQ in the range of 0.052–0.090 μg/L. This obtained LOQ was the second best in comparison to the other studies presented in Supplementary Table S6. Mashele and colleagues (Mashile et al., 2018) reported the lowest LOQ of (0.020–0.050 μg/L). Additionally, in terms of the LOD, Mashele et al. (Mashile et al., 2018) reported the lowest LOD as compared to this study. Even though a similar analytical instrument was used for analysis. Mashile and coworkers (Mashile et al., 2018) used an in-syringe micro-solid phase extraction method embedded with chitosan-activated carbon; their method was more advanced than the current study, hence the lowest LOD. In terms of the RSD, (Grover et al., 2021), (Morelli et al., 2020), (Mashile et al., 2018), and (Pasupuleti et al., 2022) reported % RSDs that were lower than <5%. The current study also reported %RSDs lower than 5%. This comparison study, presented in Supplementary Table S3, demonstrates that the biosorbent used is sufficiently sensitive and reliable compared to other adsorbents reported in the literature.

### 3.9 Reusability studies

**FIGURE 12:**
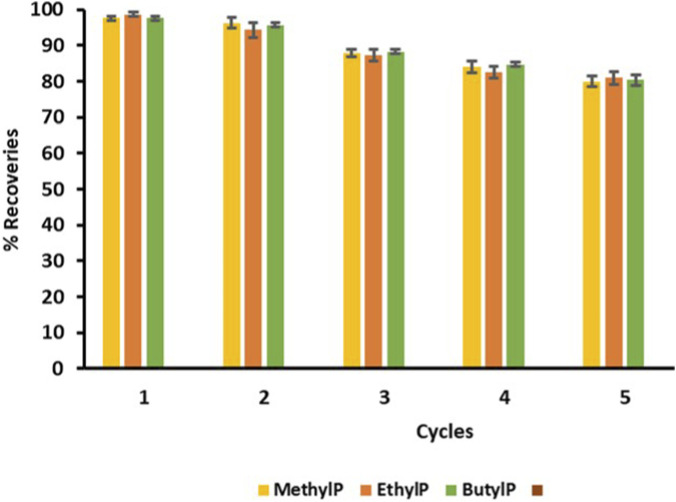
Reusability studies through extraction cycles.

The reusability evaluation revealed that 30% KOH-MAC from the avocado seed could be used for the extraction of parabens up to the 5^th^ cycle. There was no decrease in the percentage recoveries of the targeted parabens for the previous four extraction cycles. As shown in Figure 12, after the fourth cycle, a significant decline in the percentage recoveries of the parabens was identifiable. This was due to a 30% decrease in affinity towards the parabens. The decrease in affinity was caused by multiple cycles of washing, drying, and elution. Since the experiments were conducted in multiple replicates, the mean values are reported, along with standard error bars, as shown in Supplementary Figure S8. The multiple steps of washing, drying, and elution caused the biosorbent to lose the binding sites and deteriorate the pores. Nonetheless, the biosorbent was considered a reliable and stable adsorbent that possesses good reusability properties. A statistical evaluation on the t-test, for a two-tailed test, was also conducted to confirm at which reuse cycle the extraction efficiency begins to decrease. The t-test values were calculated using the t-test equation of the supplementary data. These tests were conducted on the replicates of the extraction efficiencies of the five cycles. The t-test evaluations began by formulating the null and alternative hypotheses. The null hypothesis (H_0_) was that there is no significant difference between the five extraction cycles, and the alternative hypothesis (H_a_) was that there is a significant difference between the 5 cycles. The t-test values for the three parabens were calculated and compared for the second and third cycles, as well as for the fourth and fifth cycles. These findings are shown in Supplementary Table S7. For the second and third cycles, the obtained t-values were lower than the critical t-value of 1.701 at 28 degrees of freedom, as indicated in the T distribution table. This indicated that there was no significant difference between the two cycles, and 30% KOH-MAC had sufficient strength to continue extracting the parabens. For the fourth and fifth cycles, the calculated t-values were greater than the t-critical value of 1.701 at 28 degrees of freedom. This indicated a significant difference between the fourth cycle and the fifth cycle in the extraction of the targeted parabens; therefore, the null hypothesis was rejected. This confirmed that, from the fifth extraction cycle, the extraction efficiency differs significantly from the first four cycles. This also demonstrates that the 30% KOH-MAC had lost its effectiveness by the fifth cycle, which is why there is a difference in the extraction efficiency.

## 4 Limitations and shortcomings

The optimization parameters did not include temperature, which limited our understanding of thermodynamics. This information is crucial for determining whether the process is exothermic or endothermic. Additionally, temperatures in water bodies vary seasonally, and neglecting to consider temperature can affect adsorption efficiency in both high and low-temperature conditions. Furthermore, wastewater contains numerous unknown contaminants that can interfere with the uptake of the desired contaminant.

## 5 Conclusion

The synthesized magnetic activated carbon biosorbent demonstrated significant potential for the effective extraction and preconcentration of parabens (EthylP, MethylP, and ButylP) from wastewater samples. The successful combination of Fe_3_O_4_ nanoparticles with activated carbon from avocado seed waste resulted in a biosorbent with excellent adsorption capacities and recovery rates. Characterization confirmed the successful synthesis of the desired biosorbent. Through univariate optimization, it was possible to choose the best eluent solvent and the best percentage of activation that would be effective for the extraction of the parabens in wastewater. Multivariate optimization confirmed that high percentage recoveries of the parabens can be obtained when using a pH of 6.5, a mass of adsorbent of 22.5 mg, and an eluent volume of 875 µL. The percentage recoveries of parabens in real wastewater samples were found to be in a range of 99.1%–101% for both spiked and unspiked water samples. The optimization studies established optimal conditions for the extraction process, highlighting the robust performance of the biosorbent across various parameters. Furthermore, the biosorbent’s stability and regeneration properties gradually declined in terms of preconcentration and extraction efficiency after repeated use. The material retained acceptable performance for up to four regeneration cycles, indicating its practicality and sustainability for real-water applications. Overall, this study confirms that the DMSPME-HPLC-PDA method, utilizing the 30% KOH-MAC biosorbent, is a promising approach for the preconcentration and simultaneous extraction of parabens, thereby contributing to improved wastewater treatment and environmental protection efforts.

## Data Availability

The original contributions presented in the study are included in the article/Supplementary Material, further inquiries can be directed to the corresponding author.
