# Supplementary material for: Preparation of biosorbent from avocado seeds for preconcentration and simultaneous extraction of trace parabens in environmental wastewater samples
Source: Front Chem. 2025 Nov 27;13:1688983. doi: 10.3389/fchem.2025.1688983 (PMC12696184; doi:10.3389/fchem.2025.1688983)
Supplement: Supplementary file 1 [file DataSheet1.docx]

Supplementary Material

**Preparation of biosorbent from avocado seeds for preconcentration and simultaneous extraction of trace parabens in environmental wastewater samples.**

**Aluwani Sharon Nephiphidi^1^, Rabelani Mudzielwana^2^, and Shirley Kholofelo Selahle^1*^**

^1^Department of Chemistry, Faculty of Science, Engineering and Agriculture, University of Venda, Private bag X5050, Thohoyandou, 0950, South Africa,

^2^ Department of Geography and Environmental Science, Faculty of Science, Engineering, and Agriculture, University of Venda, Private Bag X5050, Thohoyandou 0950, South Africa

*Corresponding author: Shirley.Selahle@ ac.za

Table S1: Showing various regions that have been shown to have various paraben concentrations, according to various studies.

| Country | Sample matrix | Concentration | References |
| --- | --- | --- | --- |
| South Africa | Surface water (Rivers) | ND – 13.5 µg/L | [6] |
|  | Wastewater (Influent) | ND – 84.7 µg/L | [6] |
|  | Wastewater (Effluent) | ND – 16.4 µg/L | [6] |
| Nigeria | Wastewater | 0.52 mg/L | [7] |
|  | Surface Water | 0.377 mg/L | [7] |
| China | Surface Water (Urban Rivers) | Median 8.38 ng/L | [8] |
| India | Lake water | 0.0182 mg/L | [9] |
| Switzerland | River Water (Glatt River) | Multiple parabens detected | [10] |

Table S2: Equations used to estimate parameters for kinetics models.

| **Kinetics** | **Equations** | **Parameters** |
| --- | --- | --- |
| **PFO** | $\ln( q_{e}- q_{t})=\ln q_{e}- K_{1}t$ | $q_{t}$ (mg/g) adsorption capacity at time t  $q_{e}$ (mg/g) adsorption capacity at equilibrium |
| **PSO** | $\frac{t}{q_{t}}=\frac{1}{K_{2}q_{e}^{2}}+\frac{t}{q_{e}}$ | $K_{1}$ (1·min^-1^) rate constant of the PFO model  $K_{2}$ (g·(mg·min)^–1^) rate constant of the PSO model |
|  |  | $K_{\mathrm{id}}$(mg·(${g\cdot\min}^{\frac{1}{2}}$)^–1^) intraparticle diffusion rate constant. |
| **Intraparticle diffusion** | $q_{t} =K_{\mathrm{id}}t^{\frac{1}{2}}+C$ | $C$intercept related to boundary layer thickness |

Table S3: Adsorption isotherm models of parabens onto 30%KOH-MAC

| **Isotherms** | **Equations** | **Parameters** |
| --- | --- | --- |
| **Langmuir** | $q_{e}=\frac{q_{m}K_{L}C_{e}}{1+ K_{L}C_{e}}$ | $q_{\max}$ (mg/g) maximum monolayer coverage capacity  $K_{l}$ (L/mg) Langmuir isotherm constant |
| **Freundlich** | $q_{e}=K_{F}C_{e}^{\frac{1}{2}}$ | $C_{e}$ (mg/L) Equilibrium concentration of adsorbate  $K_{f}$ (mg)^1–n^·Ln/g Freundlich isotherm constant |
|  |  |  |
| **Dubinin Radushvich** | $q_{e}=q_{s}\exp(-B\varepsilon^{2})$ | $q_{s}$ (mg/g) theoretical isotherm saturation capacity  $B$ (mol²/kJ²) activity coefficient related to mean adsorption energy  $\varepsilon$ Polanyi potential (kJ/mol) = $\mathrm{RTln}(1+ \frac{1}{C_{e}})$  mean free energy (E) = $\frac{1}{\sqrt{2B}}$ |
| **Sips** | $q_{e}=q_{m}\frac{{(K_{s}C_{e})}^{n}}{1+ {(K_{s}C_{e})}^{n}}$ | $K_{s}$ (L/mg) Sips equilibrium constant  n Heterogeneity factor |

Table S4: Analysis of variance (ANOVA) for models for %R of EthylP, MethylP, and ButylP. Statistical parameters: SS: sum of square; df: degree of freedom (df); MS: mean of the square.

| Factor | SS | df | MS | F | P |
| --- | --- | --- | --- | --- | --- |
| (1)pH (L) | 230 | 1 | 230 | 8.89 | 0.0407 |
| pH (Q) | 389 | 1 | 389 | 15.0 | 0.0179 |
| (2)EV(uL)(L) | 367 | 1 | 367 | 14.2 | 0.0197 |
| EV(uL)(Q) | 0.333 | 1 | 0.333 | 0.0129 | 0.915 |
| 1L by 2L | 5.62 | 1 | 5.62 | 0.217 | 0.666 |
| Error | 104 | 4 | 25.9 | 0.342 | 0.654 |
| Total SS | 1195 | 9 | 14.9 | 0.015 | 0.998 |
| R-Squared | 0.9913 | 0.8712 | 10.8 | 0.178 | 0.7651 |
| Adj R-Squared | 0.9805 | 0.9123 | 0.89 | 0.914 | 0.8991 |

Table S5: Parabens Concentrations (µg/L) found in the inlet and outlet of the wastewater treatment samples using the DMSPME method. *n*= 6, degrees of freedom = 5.

| Samples | MethylP | EthylP | ButylP |
| --- | --- | --- | --- |
| (WWTP 1)  Inlet | 10.68±2.20 | 9.71±0.90 | 6.10±1.29 |
| (WWTP 1)  Outlet | 4.45±1.72 | <LOD | 2.41±0.67 |
| (WWTP 2)  Inet | 17.39±0.30 | 11.81±0.67 | 9.51±0.41 |
| (WWTP 2)  Outlet | 4.39±1.6 | <LOD | 10.39±0.70 |

<LOQ: below the limit of quantification

Table S6: A comparative analysis of DMSPME-HPLC-PDA in relation to other studies reported in the literature.

| Type of Paraben | Sample matrix | Methods | Adsorbents | LOQ  (µg/L) | LOD (µg/L) | %RSD | Refs |
| --- | --- | --- | --- | --- | --- | --- | --- |
| MethylP, PropylP, and ButylP | Environmental water samples | HPLC-DAD | Chitosan-coated activated carbon |  | 6–15 | < 5 | [40] |
| MethylP, ButylP, and EthylP | River water | GC-MS | grapefruit peels activated carbon | 0.15 - 0.42 | 0.18 - 2.8 | 8-16 | [18] |
| EthylP | River water | LC-MS | Cork pellet Activated carbon | 1 - 10 | 0.3 – 3 | 3.8 | [3] |
| MethylP, ButylP, and Ethyl | Swimming pool and river | HPLC-UV | magnetic-activated carbon (coffee waste) | 0.3–0.5 | 0.1–0.3 | 2-9 | [41] |
| ButylP | Ocean water sample | HPLC | US-Fe_3_O_4_ @rGO-DSPE |  | 0.02 – 0.16 | 2-10 | [43] |
| MethylP, ButylP, EthylP, and PropylP | Environmental water sample | HPLC-PDA | Chitosan-coated activated carbon | 0.020–0.050 | 0.006–0.015 | <5% | [36] |
| MethylP, ButylP, and EthylP | Wastewater and river water | HPLC-PDA | 30% activated MAC | 0.12-0.17 | 0.25-0.049 | <5 | This study |

| EthylP | | | | | |
| --- | --- | --- | --- | --- | --- |
|  | Cycle 1 | Cycle 2 | Cycle 3 | Cycle 4 | Cycle 5 |
| N | 10 | 10 | 10 | 10 | 10 |
| mean | 97.67 | 96.52 | 96.61 | 84.00 | 81.98 |
| Spooled |  | 0.026 | | 0.093 | |
| DF |  | 28 | | 28 | |
| t-test value |  | 0.045 | | 1.450 | |
| MethylP | | | | | |
|  | Cycle 1 | Cycle 2 | Cycle 3 | Cycle 4 | Cycle 5 |
| N | 10 | 10 | 10 | 10 | 10 |
| mean | 98.00 | 96.95 | 97.00 | 92.00 | 90.95 |
| Spooled |  | 0.031 | | 0.045 | |
| DF |  | 28 | | 28 | |
| t-test value |  | 0.091 | | 1.966 | |
| ButylP | | | | | |
|  | Cycle 1 | Cycle 2 | Cycle 3 | Cycle 4 | Cycle 5 |
| N | 10 | 10 | 10 | 10 | 10 |
| mean | 94.00 | 92.01 | 91.89 | 85.00 | 83.98 |
| Spooled |  | 0.026 | | 0.093 | |
| DF |  | 28 | | 28 | |
| t-test value |  | 0.015 | | 2.094 | |

N: is the total number of samples

DF: Degree of freedom

Degree of freedom (DF) = N_1_+N_2_ – N_t_

= 10 + 10 -2

= 28, t critical = 1.701

Equation used to calculate the t-test value:

S_pooled =_ $\sqrt{\frac{\mathsf{\Sigma}\left( x1-\bar{x} \right)2 -\left( x2-\bar{x} \right)2}{N1+N2-Nt}}$ ……………… (5)

t = $\frac{X1-X2}{Spooled\sqrt{\frac{N1+N2}{N1\times N2}}}$…………………………. (6)


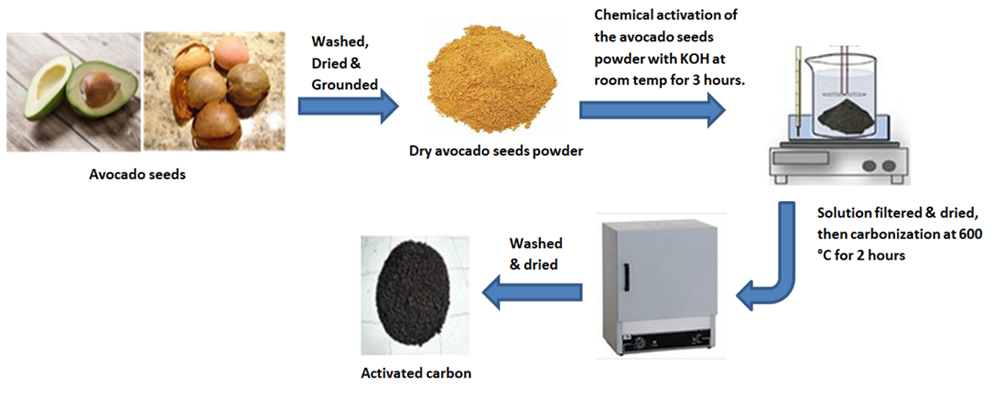


Figure S1: FTIR spectra of the avocado seeds powder


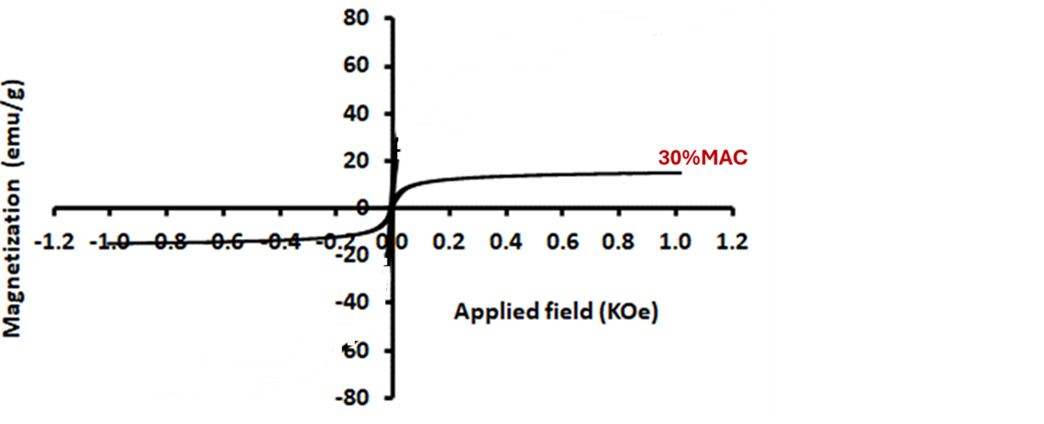


Figure S2: Vibrating sample magnetometer plot of the synthesized 30%MAC.


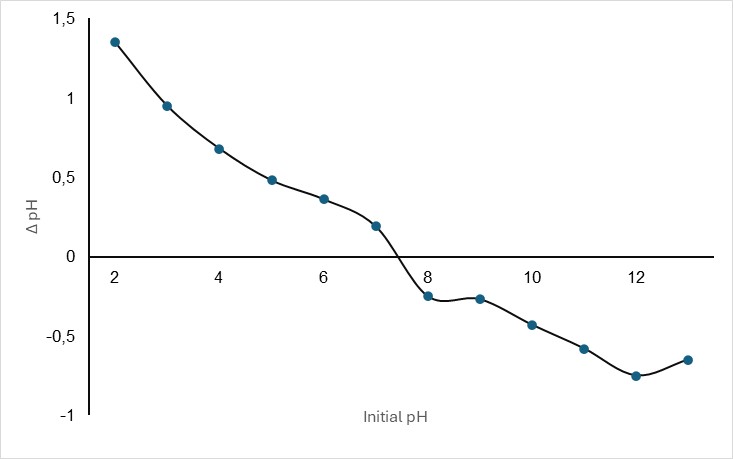


Figure S3: Graph showing the point of zero charge of the MAC 30%


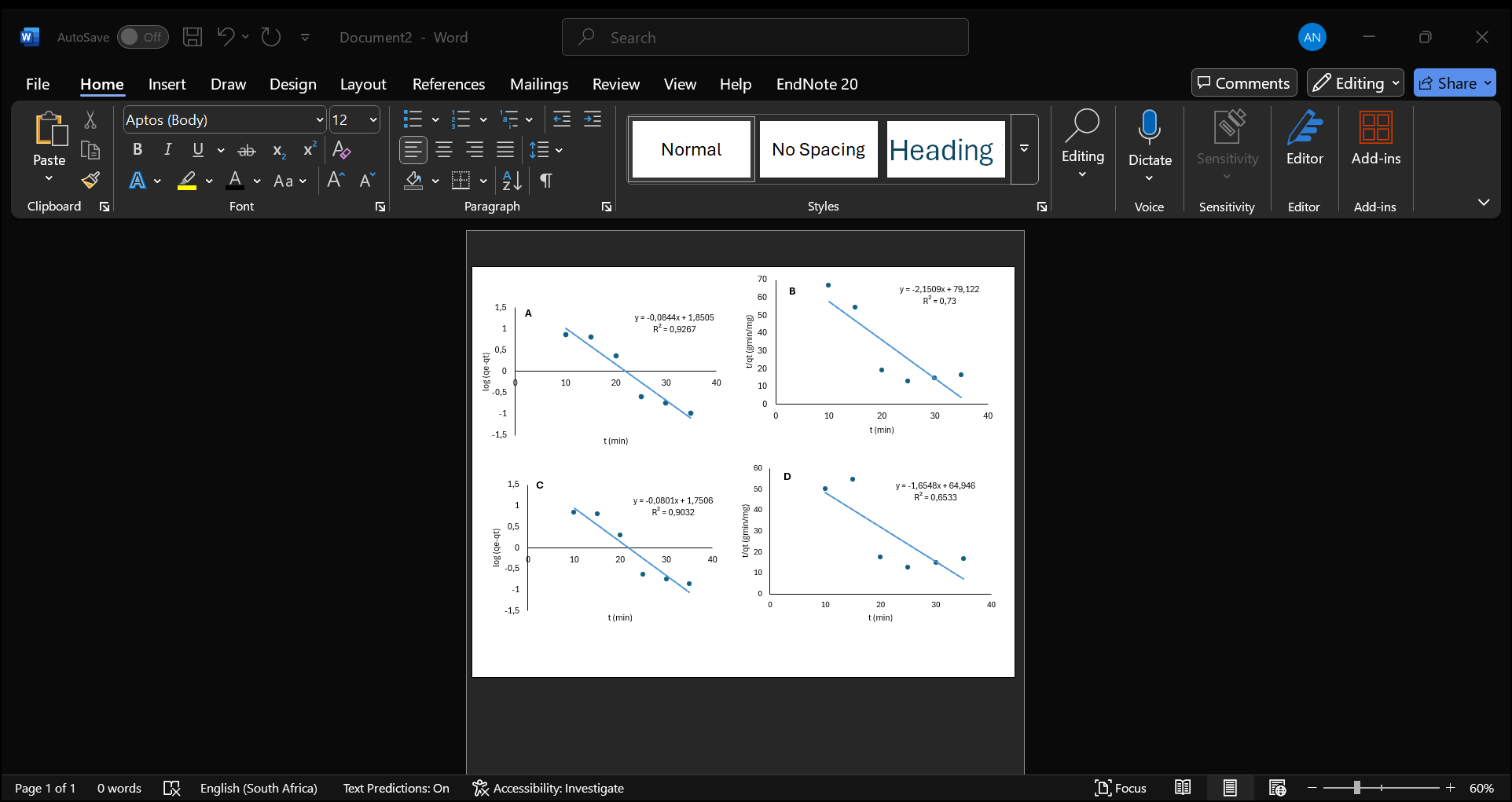


Figure S4: EthylP linear plots according to (A) the pseudo-first-order,(B) the pseudo-second-order kinetic models, and MethylP linear plots according to (C) the pseudo-first-order and (D) the pseudo-second-order kinetic models.


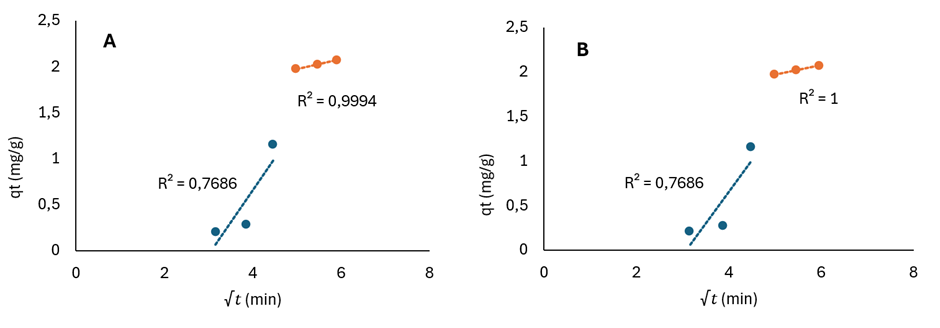


Figure S5: (A) EthylP and (B) MethylP plots according to the Intra particle diffusion kinetic models.


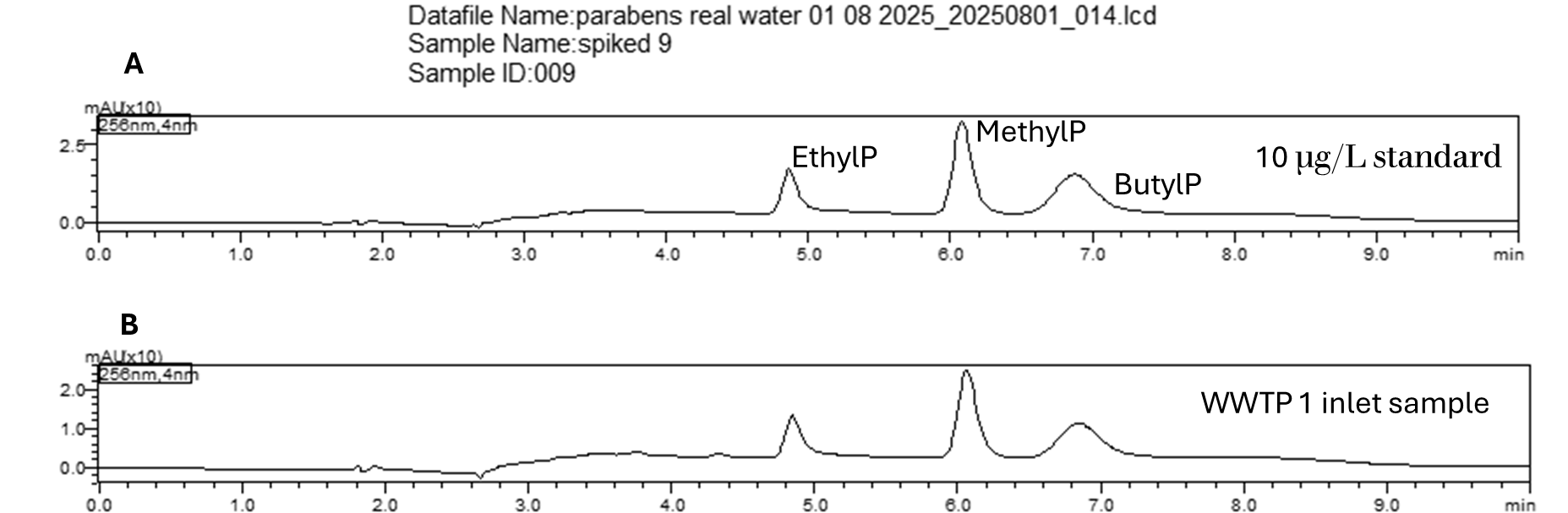


Figure S6: Chromatograms of 10 µg/L standard and of the real wastewater sample for WWTP 1 inlet


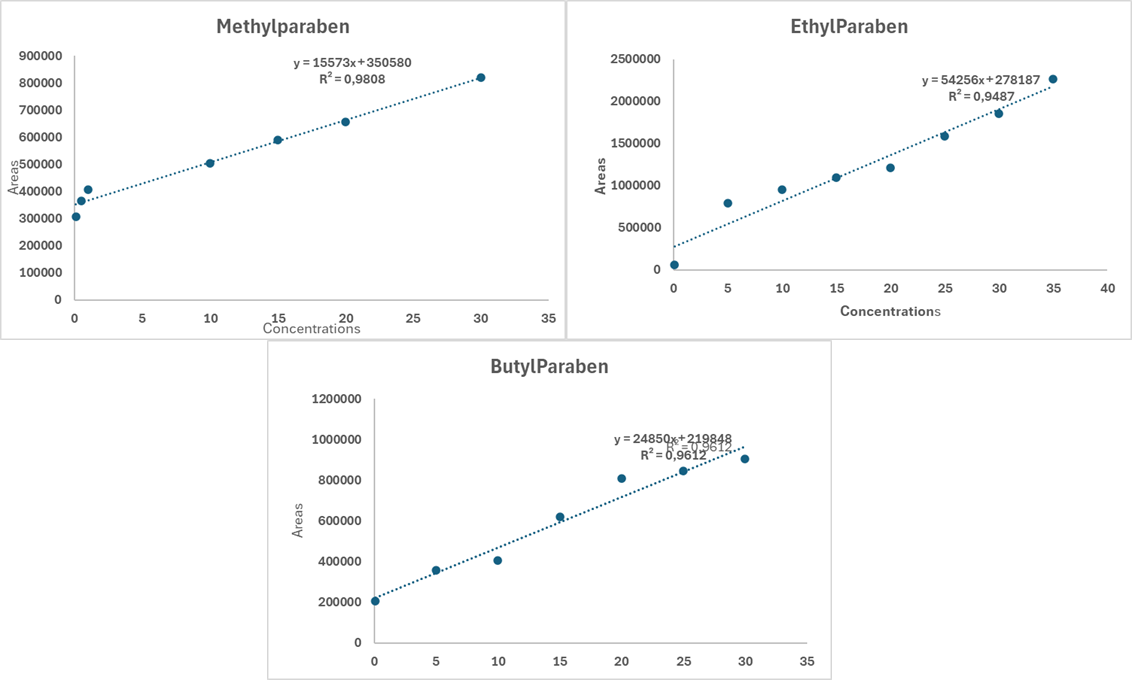


Figure S7: Calibration Curves.
